# Supplementary material for: Evaluation of an Antibiotic Susceptibility Testing Method on Enterobacterales-Positive Blood Cultures in Less Than 8 h Using the Rapid Mueller-Hinton Diffusion Method in Conjunction with the SIRscan 2000 Automatic Reading Device
Source: Microorganisms. 2022 Jul 8;10(7):1377. doi: 10.3390/microorganisms10071377 (PMC9320083; doi:10.3390/microorganisms10071377)
Supplement: Supplementary file 1 [file microorganisms-10-01377-s001.zip › microorganisms-1774053-supplementary.pdf]

Supplementary Table S1: Imipenem. meropenem. cefotaxime and ceftazidime's MICs of 39 select *Enterobacterales* isolates evaluated using E-test (BioMerieux®).

		MIC E-test (mg/L)			
Bacteria	Phenotypic mechanism	imipenem	meropenem	cefotaxime	ceftazidime
E. coli	carbapenemase	16 (R)	32 (R)	> 32 (R)	> 256 (R)
E. coli	carbapenemase	0.75 (S)	0.25 (S)	1.5 (I)	0.38 (S)
E. coli	carbapenemase	0.5 (S)	0.75 (S)	1 (S)	0.5 (S)
K. pneumoniae	carbapenemase	12 (R)	4 (I)	> 32 (R)	> 256 (R)
K. pneumoniae	carbapenemase. ESBL	32 (R)	32 (R)	> 32 (R)	> 256 (R)
K. pneumoniae	carbapenemase. ESBL	32 (R)	32 (R)	> 32 (R)	> 256 (R)
E.cloacae	carbapenemase. ESBL	2 (S)	2 (S)	> 32 (R)	> 256 (R)
C. freundii	carbapenemase. ESBL. AmpC hyperproduction	2 (S)	0.75 (S)	> 32 (R)	> 256 (R)
C. freundii	carbapenemase. ESBL. AmpC hyperproduction	1.5 (S)	2 (S)	> 32 (R)	32 (R)
E.cloacae	carbapenemase. ESBL. AmpC hyperproduction	(S)	0.75 (S)	> 32 (R)	48 (R)
K. oxytoca	ESBL	0.125 (S)	0.016 (S)	3 (R)	32 (R)
E. coli	ESBL	0.19 (S)	0.016 (S)	> 32 (R)	3 (I)
K. pneumoniae	ESBL	0. 38 (S)	0.75 (S)	> 32 (R)	> 256 (R)
E. coli	ESBL	0.25 (S)	0.023 (S)	> 32 (R)	6 (R)
E. coli	ESBL	0.19 (S)	0.032 (S)	> 32 (R)	32 (R)
E. coli	ESBL	0.19 (S)	0.023 (S)	> 32 (R)	2 (I)
K. pneumoniae	ESBL	0.19 (S)	0.047 (S)	> 32 (R)	192 (R)
E. coli	ESBL	0.125 (S)	0.023 (S)	> 32 (R)	0.75 (S)
E. coli	ESBL	0.125 (S)	0.023 (S)	> 32 (R)	0.75 (S)
E. coli	ESBL	0.19 (S)	0.023 (S)	32 (R)	2 (I)
E. coli	ESBL	0.19 (S)	0.032 (S)	> 32 (R)	4 (I)
E. coli	ESBL	0.19 (S)	0.047 (S)	> 32 (R)	48 (R)
E. cloacae	ESBL. AmpC hyperproducer	0.25 (S)	0.064 (S)	> 32 (R)	> 256 (R)
E. cloacae	AmpC hyperproduction	0.25 (S)	0.047 (S)	12 (R)	0.5 (S)
E. cloacae	AmpC hyperproduction	0.38 (S)	0.047 (S)	> 32 (R)	32 (R)
E. coli	AmpC hyperproduction	0.38 (S)	0.032 (S)	> 32 (R)	128 (R)
E. coli	AmpC hyperproduction	0.25 (S)	0.032 (S)	4 (R)	6 (R)
S. marcescens	AmpC hyperproduction	0.38 (S)	0.047 (S)	24 (R)	0.75 (S)
K. oxytoca	plasmidic cephalosporinase	0.25 (S)	0.047 (S)	3 (R)	4 (I)
P. mirabilis	plasmidic cephalosporinase	0.25 (I)	0.5 (S)	> 32 (R)	24 (R)
E. coli	plasmidic cephalosporinase	0.19 (S)	0.016 (S)	4 (R)	4 (I)
E. coli	plasmidic cephalosporinase	0.19 (S)	0.125 (S)	> 32 (R)	> 256 (R)

K. pneumoniae	plasmidic cephalosporinase	0.75 (S)	0.064 (S)	16 (R)	12 (R)
K. pneumoniae	plasmidic cephalosporinase	0.25 (S)	0.032 (S)	> 32 (R)	32 (R)
K. pneumoniae	Penicillinase hyperproduction	0.125 (S)	0.012 (S)	0.38 (S)	1 (S)
K. pneumoniae	Penicillinase hyperproduction	0.19 (S)	0.023 (S)	0.094 (S)	6 (R)
P. mirabilis	oxacillinase	0.25 (I)	0.032 (S)	0.032 (S)	0.19 (S)
E. coli	resistance-to-inhibiteur TEM	0.125 (S)	0.016 (S)	0.094 (S)	0.25 (S)
E. coli	oxacillinase	0.25 (S)	0.032 (S)	0.5 (S)	0.094 (S)

S = susceptible, I = intermediate and R = resistant.
